# Drug Release Studies from Caesalpinia pulcherrima Seed Polysaccharide

**Published:** 2011

**Authors:** Somasundaram Jeevanandham, Duraiswamy Dhachinamoorthi, Kothapalli Bannoth Chandra Sekhar

**Affiliations:** a *Santhiram College of Pharmacy, Nandyal.*; b *QIS College of Pharmacy, Ongole.*; c *Jawaharlal Nehru Technological University, Anantapur, Andhrapradesh, India-515002.*

**Keywords:** Caesalpinia pulcherrima, Kernel powder, Natural gums, Polysaccharide, Drug release.

## Abstract

This study examines the controlled release behavior of both water-soluble (acetaminophen, caffeine, theophylline and salicylic acid) and water insoluble (indomethacin) drugs derived from Caesalpinia pulcherrima seed Gum isolated from Caesalpinia pulcherrima kernel powder. It further investigates the effect of incorporating diluents such as microcrystalline cellulose and lactose on caffeine release. In addition the effect the gum’s (polysaccharide) partial cross-linking had on release of acetaminophen was examined. Applying the exponential equation, the soluble drugs mechanism of release was found to be anomalous. The insoluble drugs showed a near case II or zero order release mechanism. The rate of release in descending order was caffeine, acetaminophen, theophylline, salicylic acid and indomethacin. An increase in the release kinetics of the drug was observed on blending with diluents. However, the rate of release varied with the type and amount of blend within the matrix. The mechanism of release due to effect of diluents was found to be anomalous. The rate of drug release decreased upon partial cross-linking and the mechanism of release was found to be of super case II.

## Introduction

Hydrophilic matrices are an interesting option when developing an oral sustained-release formulation. They can be used for the controlled release of both water-soluble and water-insoluble drugs. The release behaviour of drugs varies with the nature of the matrix, which is the complex interaction of the swelling, diffusion and erosion process (1). The release of drugs from such matrices can be controlled through their physical properties by using the correct choice of gelling agent and setting up the conditions to allow fabrication (2). From among hydrophilic polymers, polysaccharides are the material of choice due to their nontoxicity and acceptance by the regulating authorities (3). Polysaccharides such as cellulose ethers (4), xanthan gum (5), scleroglucan (6), locust bean gum (7) and gaur gum (8) are some of the natural polysaccharides that have been evaluated within the hydrophilic matrix for the drug delivery system. Although Caesalpinia pulcherrima seed polysaccharide (CPSP) is used as an ingredient in food preparation and pharmaceuticals it has not been evaluated with the view to being used as a hydrophilic drug delivery system. CPSP is a galactoxyloglucan isolated from the seed kernel of Caesalpinia pulcherrima. It possesses properties such as high viscosity, broad pH tolerance and adhesivity (9). These properties have led to it being used as a stabilizer, thickener, gelling agent and binder in both the food and pharmaceutical industries. In addition to these known properties of CPSP other important ones have been recently identified. These include non-carcinogenicity (10), mucoadhesivity, biocompatibility (11), high drug holding capacity (12) and high thermal stability (13). These findings have led to its application as an excipient in the hydrophilic drug delivery system (11-12). As CPSP is an important excipient, the present study was undertaken to elucidate the release kinetics of both the water-soluble and water insoluble form of the drug from this matrix. In order to predict and correlate the release behaviour of the drugs from the hydrophilic matrix it is necessary to fit it into a suitable model. The commonly adopted model for understanding such behaviour from hydrophilic matrices is the simple exponential equation (14). This model facilitates the understanding of the mode of release such as whether the release is due only to diffusion and/or erosion. This model was used for this study.

## Experimental

*Materials*

Caesalpinia pulcherrima seeds were collected from the Kurnool district Andhrapradesh (India). Acetaminophen and caffeine were obtained as a gift sample from Tablets India Limited, Chennai. Salicylic acid was acquired from Qualigens (India). Indomethacin and theophylline anhydrous were purchased from the Sigma Chemicals Company. Microcrystalline cellulose, lactose monohydrate, magnesium stearate were purchased from Central Drug House (India). Absolute ethanol, diethyl ether, petroleum ether, glacial acetic acid, epichlorohydrin and acetone were acquired from Qualigens (India). The sodium hydroxide was manufactured by E-Merck (India). All the chemicals used were of analytical grade.

*Isolation of CPSP*

CPSP was prepared in three batches following the method set out by Rao *et al*.(8,15) on a laboratory scale. To 20 g of Caesalpinia pulcherrima kernel powder 200 mL of cold distilled water was added forming a slurry. The slurry was poured into 800 mL of boiling distilled water. The solution was boiled for 20 min under constant stirring within a water bath. The resulting thin clear solution was kept overnight to allow the majority of the containing proteins and fibers to settle out. The solution was then centrifuged at 5000 rpm for 20 min. The supernatant was separated and poured into double the volume of absolute ethanol via continuous stirring. The resulting product was then pressed between felt. The precipitate was washed with absolute ethanol, diethyl ether and petroleum ether, and then dried at 50-60°C under a vacuum. The dried material was ground and sieved to obtain a range of different-sized particle granules. The particles ranged 150-75 microns in size were used for the preparation of the tablets.

*Characterization of CPSP by C13 NMR and X-ray diffraction*

*N.M.R. spectroscopy*

The C^13^ N.M.R spectrum was recorded for the CPSP solution in D_2_O. The sample was dissolved by heating.

*X-ray diffraction*

The diffraction pattern of the powdered CPSP sample was recorded with an X-ray diffractometer (CECRI, Tuticorin). X-ray diffraction was performed at room temperature (30°C) with the aid of a diffractometer; the target, Cu (λ = 1.54 Å), filter, Ni; Voltage, 40 kV; current 30 mA; time constant 10 mm/s and scanning rate 2°/min were measured from 10-35° at a full scale of 200.

*Cross-linking of CPSP*

CPSP was partially cross linked with epichlorohydrin (16). 10 g of CPSP (soaked in water) and sodium hydroxide (50 mL, 1 N, 54°C) was mixed with a glass rod. After homogenization (15 min), 0.5 mL epichlorohydrin (6 g/100 g of CPSP) was slowly added by continuous homogenization (15 min). The gel was then neutralized with acetic acid and washed 3 times through a sintered glass filter using a solution of water/acetone (60 : 40 v/v). During the final step of the process, the resulting solid gel was placed over a filter and washed with pure acetone. The resulting polymer was air dried at room temperature for 72 h and then stored in an airtight container. After granulation, granular fractions ranging from 75 to 250 microns were used for the preparation of the tablets. In addition cross-linked polysaccharide was prepared in three batches.

*Preparation of tablet*

The total weight of the tablets (without magnesium stearate) was 250 mg for a drug: polymer ratio of 1 : 4 and 300 mg for drug : polymer ratio of 1 : 2. The ingredients (Table 1) were mixed in mixer for 5 min before and after the addition of magnesium stearate (lubricant). The tablets were prepared using a single-punch hand operated tablet machine (Cadmach) fitted with flat-faced punches at a compression pressure of 5 Tons for 30 sec. The diameter of the tablet was 13 mm and this was kept constant through out the experiment.

**Table 1:** Formulations of various Caesalpinia pulcherrima seed polysaccharide matrices

**Ingredients**	**Drug type (mg/tablet)**	**Cross linker (mg/tablet)**	**Diluents (mg/tablet)**
Drug Substance^a^	50	50/100	50
CPSP^b^	200	0	180/160/140/120/100/80
Cross-linked CPSP	0	200	0
Lactose/MC^c^	0	0	20/40/60/80/100/120
Magnesium Stearate	2.5	2.5/3	2.5

^a^Caffeine/Acetaminophen/Theophylline/Salicylic acid/Indomethacin, ^b^Caesalpinia pulcherrima Seed Polysaccharide (CPSP), ^c^Microcrystalline Cellulose (MC)

*Equilibrium swelling study*

The equilibrium swelling volume (16) of the partially cross-linked CPSP powder and tablets was measured in water at 37°C. The drug free tablets each weighing 250 mg (or 250 mg of powder) were placed in a 25 mL graduated cylinder to which 10 mL of water was added. After 48 h, the equilibrium swelling volume was read directly as the volume of the gel bed. The swelling was expressed as swollen volume per unit weight of initial dry material (mL/g).

*In-vitro drug release study*

Single face release experiments were performed at 37°C. The sample holder was immersed in 900 mL-distilled water for caffeine, acetaminophen, theophylline, salicylic acid and in phosphate buffer with pH 7.2 for indomethacin. The sink condition was followed for the entire experiment, as the volume of dissolution medium was above 10 times the solubility of the drugs within the dissolution medium. Agitation of 100 rpm was provided and the concentration of drug in the dissolution medium was measured as a function of time. The concentration of caffeine, acetaminophen, theophylline, salicylic acid and indomethacin was determined by monitoring the UV absorbance of the dissolution medium at 273, 242, 271, 297 and 318nm respectively (Table 2). The experiments were repeated for each batch and average values were recorded.

**Table 2:** List of model drugs used for preparation of matrix tablet

**Drug Type**	**Solubility in water** **at 37°C (mg/mL)**	**Detection** **wave length (nm)**
Caffeine anhydrous	37.0	273
Acetaminophen	18.9	242
Theophylline Anhydrous	9.9	271
Salicylic acid	3.1	297
Indomethacin	0.9	318

^a^ Phosphate buffer pH 7.2

*Model used for analysis of drug release kinetics*

The dissolution data was fitted according to the well-known exponential equation (14), which is often used to describe the drug release behaviour from polymeric systems.

M_t_/M_∞_ = k t^n^(1)

Where M_t_/M_∞_ is the fractional release of the drug, ‘t’ is the release time, ‘K’ is a constant incorporating the structural and geometric characteristic of the release device (tablets) and n is the release exponent indicative of the mechanism of release. Table 3, shows an analysis of the diffusional release mechanism obtained by varying the n values (17). The n values used for the analysis of the drug release mechanism from the tablets were determined from log (M_t_/ M_∞_) vs. log (t) plots.

**Table 3:** Variation of n values with mechanism of diffusion

**n**	**Mechanism**	**dM**_t_ **/dt dependence**
0.5	Fickian diffusion	t^-0.5^
0.5 < n > 1.0	Anomalous diffusion	t^n-1^
1.0	Case II transport	Zero order
n > 1.0	Super case II transport	t^n-1^

## Results and Discussion

*Characterization of CPSP*

**Figure 1:**
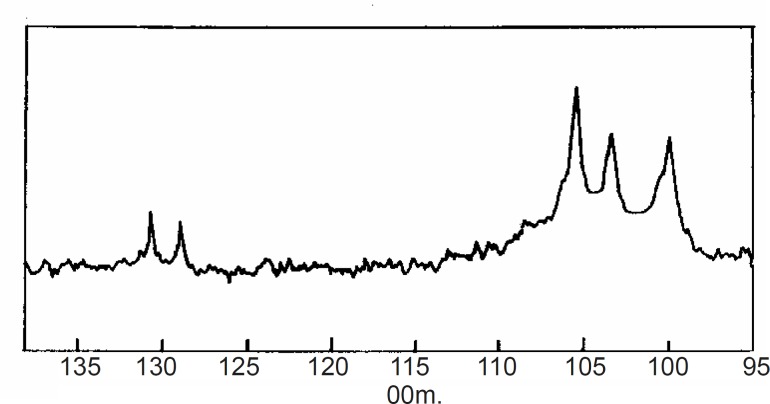
^13^C-N.M.R. spectrum of Caesalpinia pulcherrima seed polysaccharide

^13^C N.M.R: The^ 13^C N.M.R spectrum of CPSP is shown in Figure 1. The spectrum shows C-1 signals at 105.4, 103.4 and 100.0 ppm that are assigned to galactose, glucose and xylose residues respectively. The result complies with the reported values (18).

**Figure 2:**
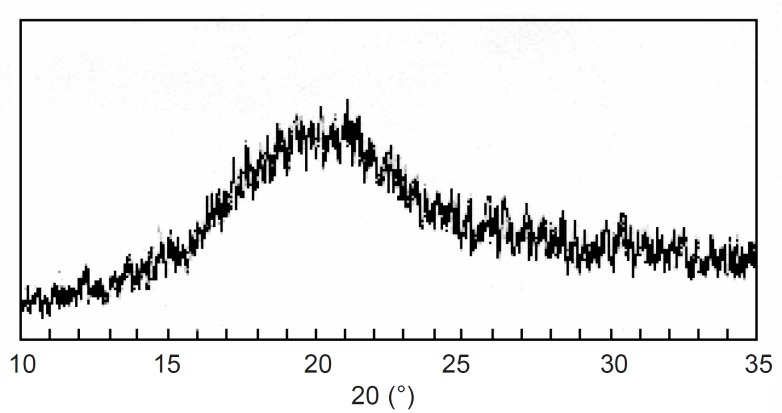
X-ray diffraction pattern of Caesalpinia pulcherrima seed polysaccharide

X-ray diffraction analysis: The X-ray diffraction pattern (Figure 2) of CPSP did not show any characteristic peak, which indicates that the structure is completely amorphous.

The result confers with the X-ray diffraction study of Caesalpinia pulcherrima xyloglucan (19). The results show that the isolated polysaccharide has similar behavior with that reported by others. Therefore, the polysaccharide isolated can be used in the following study.

*Effect of solubility of drug*

**Figure 3:**
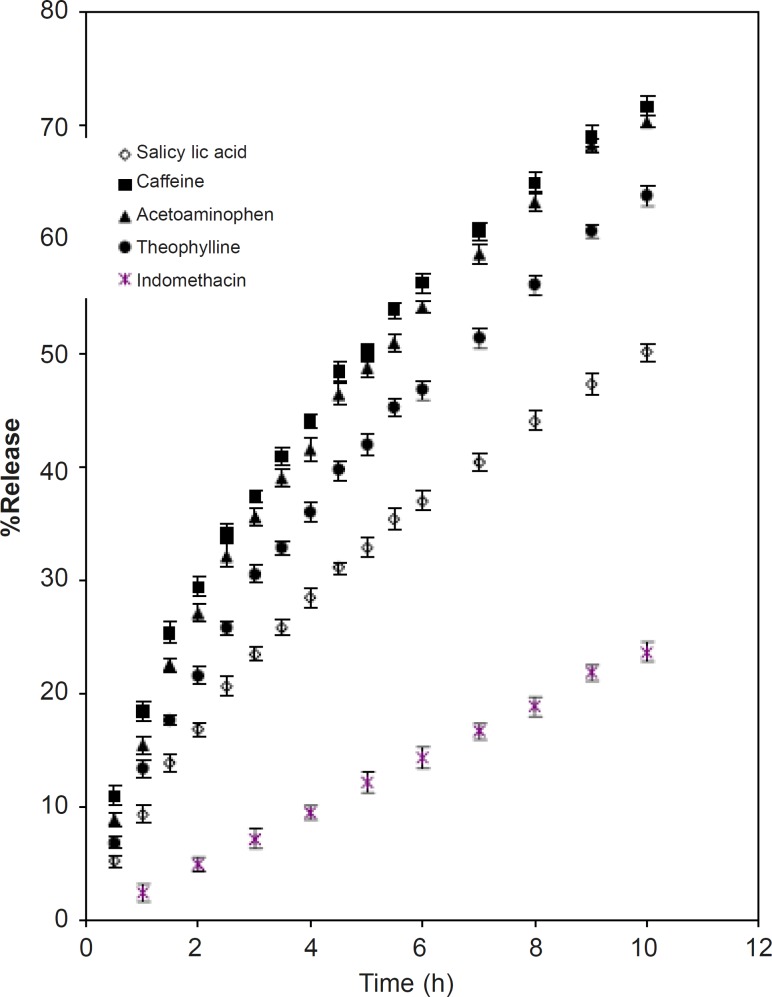
Release profile of drugs of different solubility from CPSP tablets (mean ± SD; n = 3)

The release of the drug depends not only on the nature of the matrix but also on the solubility of the drug. As such the release of drugs with different solubility parameters such as caffeine, acetaminophen, theophylline, salicylic acid and indomethacin were studied (Table 2). The intrinsic dissolution of the drugs within the dissolution medium was determined. The procedure outlined by Tarara *et al. *(20) was followed. 1 g of the drugs was placed in 10 mL of dissolution medium and kept on a shaker at 37°C for 42 h. 5 mL of the resulting solution was centrifuged at 5000 rpm for 15 min. The supernatant collected was then passed through a Millipore filter. The absorbance values were measured allowing the respective absorbance and solubility values to be calculated (Table 2). The rate of drug release from the matrices (Figure 3) were in decreasing order of the solubility parameters. The mechanism of release of soluble drugs was anomalous (n > 0.5), while indomethacin (water insoluble drug) showed behavior of near case II or zero order release (Table 4).

**Table 4:** The n value of the formulations containing drug type with D : P ratio of 1 : 4 and cross linker with D:P ratio of 1: 2 and 1 : 4

**Formulation**	**n**
**Drug type**	
Caffeine	0.60
Acetaminophen	0.66
Theophylline	0.71
Salicylic acid	0.73
Indomethacin	0.98
**Cross- linker**	
(1:2)	1.24
(1:4)	1.25

 This indicates that release is controlled by both diffusion and erosion phenomena. The latter dominates the release of the drug as its solubility in water decreases and vice versa (20). About 50% of the overall loaded drug is released 5, 5.5, 7 and 10 h for caffeine, acetaminophen, theophylline and salicylic acid respectively. The total release percentage of the soluble drugs in the space of 5 hours decreases from 50% to 32% as the solubility of drug in water decreases. The total release of indomethacin in the first 5 hours is about 10% of total tablet load. The rate of drug release decreases with the decrease in the solubility of the drugs. This is because water dissolves the drug at the surface to begin with and then penetrates the matrix via pores causing the polymer to gel. The dissolved drug is then released via diffusion through the gel and finally the release rate falls as the water reaches the center due to a decreased drug concentration of less than its solubility (1, 21). The solubility of indomethacin in the aqueous medium (phosphate buffer) is very low. Due to the slow erosion of the matrix and low solubility the amount of drug released is also limited. The value of n varies from anomalous to near zero order as the solubility of the drugs decrease (Table 4).

*Effect of diluents*

**Figure 4:**
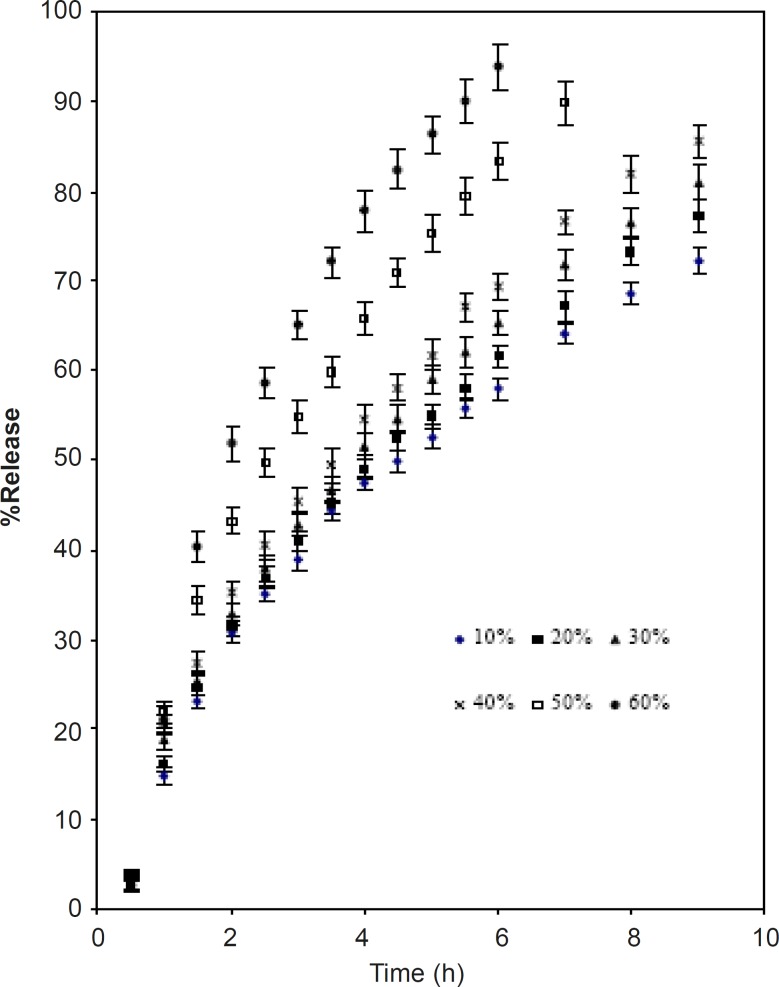
Effect of replacing CPSP with lactose on release of caffeine (mean ± SD; n = 3).

**Figure 5:**
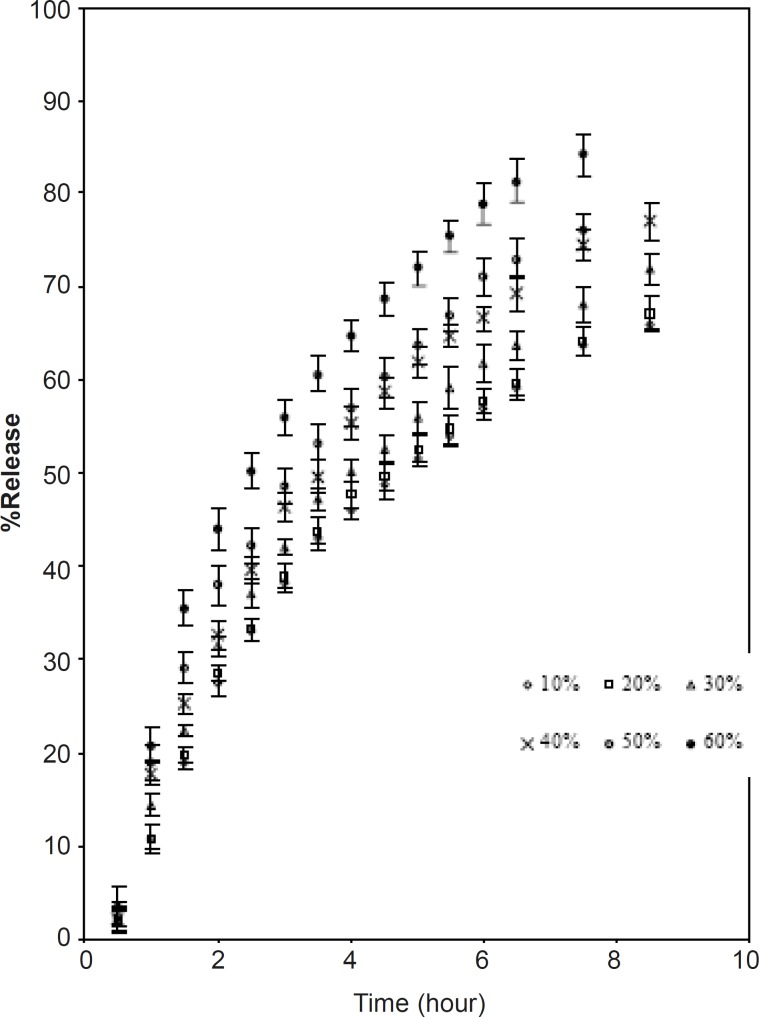
Effect of replacing CPSP with microcrystalline cellulose on release of caffeine (mean ± SD; n = 3).

Figure 4 and 5 show the effect of diluents. Two materials were chosen for this purpose namely lactose and microcrystalline cellulose. The former is water-soluble while the latter is relatively hydrophobic. These two were separately blended along with CPSP and caffeine (Table 1).

The mechanisms of release of caffeine from the blends were found to be anomalous (Table 5)

As the percentage of diluents increased, the kinetics of release also increased. This may be due to the structural reorganization of the hydrophilic polysaccharide matrix (3, 22, 23, 24). The lactose being water soluble undergoes dissolution which may result in a reduction in the tortuosity and/or gel strength of the polymer. The T_50_ value of lactose and microcrystalline cellulose were almost identical up to 40%. Above 40% the rate of release was faster in the case of lactose (Table 5). The slow release could be due to a reported interaction between Caesalpinia pulcherrima seed polysaccharide and microcrystalline cellulose (19).

**Table 5:** The n value of formulations containing D : P ratio of 1: 4 when replacing the polymer with different amount of lactose and microcrystalline cellulose

**% Replacement**	**n**	**t**_50_
**Lactose**		
0%	0.60	5.0
10%	0.59	4.5
20%	0.60	4.0
30%	0.61	3.75
40%	0.61	3.5
50%	0.61	2.5
60%	0.59	2.0
**MC**		
0%	0.60	5.0
10%	0.59	4.5
20%	0.56	4.5
30%	0.56	4.0
40%	0.58	3.5
50%	0.57	3.15
60%	0.52	2.5

*The effect of partial cross-linking of matrix*

**Figure 6:**
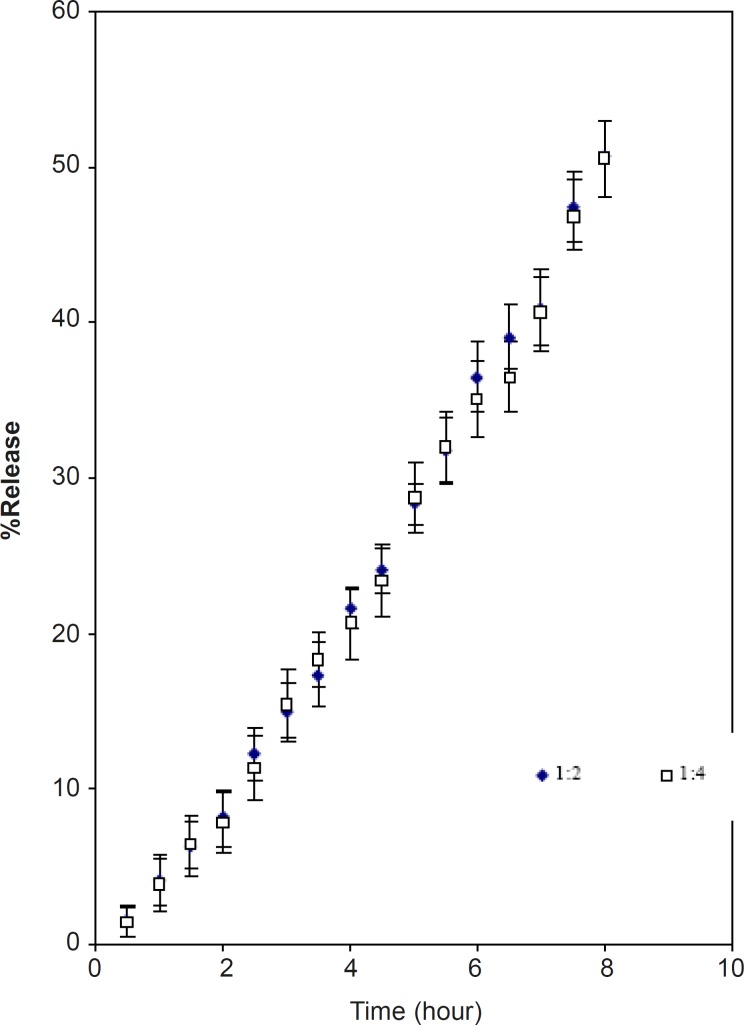
The release profile of acetaminophen from cross linked CPSP tablets (mean ± SD; n = 3).

The partially cross-linked CPSP powder and tablet had an equilibrium swelling volume of 22 mL/gm and 12 mL/gm respectively. This shows that intergranular hydrogen bonds exist within the tablets due to compression like that of cross linked amylase (16). The mechanism of drug (acetaminophen) release from the two formulations of cross-linked CPSP was found to be of super case II (Table 3) and the dissolution T_50 _value for drug was 8 h (Figure 6). The release can be sustained at a constant rate for a longer period than with uncross-linked material. Drug loading had no effect on the percent of release (Figure 6). The slow rate of drug release could be due to slow water penetration due to the presence of numerous intergranular hydrogen bonds and the gel barrier (16). This illustrates that by controlling the degree of cross-linking; the release kinetics can be optimized to achieve the desired design.

## Conclusion

Caesalpinia pulcherrima seed polysaccharide can be used for the controlled release of both water-soluble and water insoluble drugs. Zero order release can be achieved by removing a scarcely soluble drug such as indomethacin from CPSP. The rate of release can be controlled by using suitable diluents such as lactose and microcrystalline cellulose. For water-soluble drugs the release amount can also be controlled by partially cross linking the matrix. The extent of release can be varied by controlling the degree of cross-linking.
